# Glioblastoma multiforme of spinal cord – Case series in a tertiary cancer centre

**Published:** 2021-11-29

**Authors:** Aswin Nagarajan, Ramya Ravichandar

**Affiliations:** ^1^Department of Radiation Oncology, Cancer Institute (WIA), Chennai, Tamil Nadu, India; ^2^Department of Pharmacology, Sree Balaji Medical College and Hospital, Chennai, Tamil Nadu, India

**Keywords:** glioblastoma multiforme, spinal cord, radiotherapy, chemotherapy

## Abstract

**Background and Aim:**

Primary spinal cord glioblastoma multiforme (GBM) is a rare clinical condition and is often associated with a dismal prognosis. The standard treatment is maximal safe surgery followed by adjuvant radiotherapy and chemotherapy. Despite such aggressive treatments, the median survival is estimated to be around 15 months in several studies. We report three patients with primary spinal GBM who received treatment in our institute from 2012 to 2019. Among the three, one patient is on long-term follow-up with no evidence of disease, another patient succumbed to the illness and the third patient is having stable disease.

**Relevance for Patients:**

Although primary spinal GBM is usually associated with a dismal prognosis, our case series shows a subset of patients will have a favorable outcome with the protocol treatment.

## 1. Introduction

Although the most common brain tumor in adults is glioblastoma multiforme (GBM), it is very uncommon in the spinal cord accounting for only 4-8% all primary central nervous system lesions [1]. It most commonly occurs during the second or third decade of life with more common predilection toward the thoracic spine [2]. The standard treatment is maximal safe surgical resection followed by post-operative radiotherapy usually with concurrent chemotherapy and adjuvant chemotherapy [3]. Since there is a high chance of seeding of these tumors into the cerebrospinal fluid (CSF), CSF cytology along with magnetic resonance imaging (MRI) of the entire neuroaxis including the brain is generally advised in this condition. We report three patients with primary spinal GBM who underwent treatment in our institute from 2012 to 2019. Among the three, one patient is on long-term follow-up with no evidence of disease, another patient succumbed to the illness, and the third patient is with stable disease.

## 2. Case Presentation

### 2.1. Case 1

**Figure 1:**
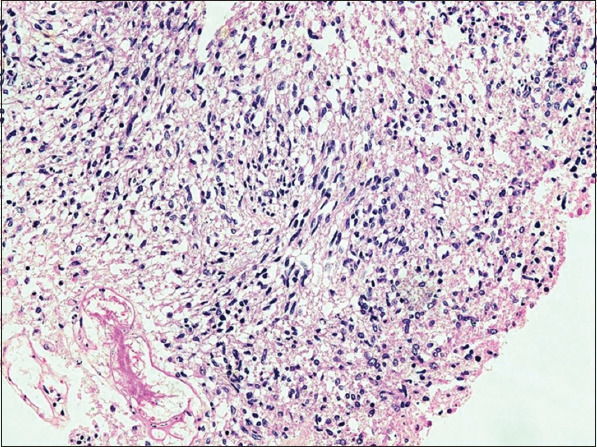
The microscopic appearance of glioblastoma multiforme

A 32-year-old male with no known comorbidities, without any history of addictions was evaluated for intractable neck pain in January 2012. MRI of the entire neuroaxis revealed a well-defined intramedullary lesion measuring 5.1×2.9 cm at C1-C4 level in the spine. Clinical examination revealed no evidence of neurological deficit. The sensory system was normal. There were no bowel or bladder disturbances. Deep tendon reflexes and the plantar reflex were normal. He underwent laminectomy and radical excision of the lesion. The postoperative period was uneventful. The postoperative histopathological examination (HPE) was suggestive of GBM (Figure 1). The CSF cytology was normal. The postoperative MRI was suggestive of intramedullary irregular rim at C2-C3 level with significant reduction in volume. His post-operative neurological status was stable. He then received adjuvant radiotherapy using conformal technique of a total dose of 4400 cGy along with concurrent temozolomide (75 mg/m^2^) along with corticosteroids, followed by 6 cycles of adjuvant temozolomide (150 mg/m^2^). The patient is still on follow-up with annual MRI with no evidence of disease or neurological deficit. The disease-free survival for this patient is 96 months. To the best of our knowledge, this patient has the longest survival quoted in the literature.

### 2.2. Case 2

**Figure 2:**
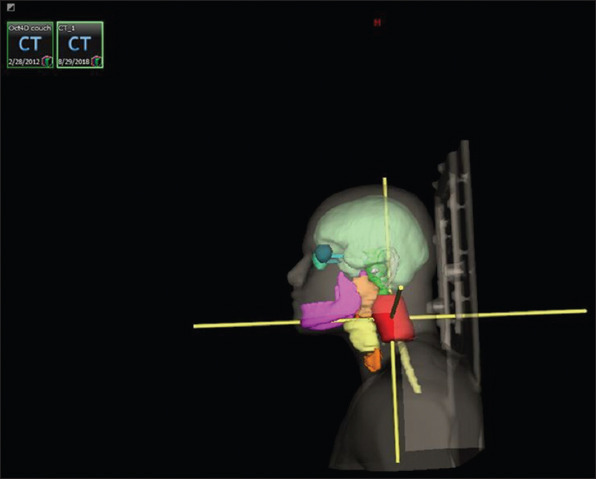
Three-dimensional plan of cervical cord lesion

**Figure 3:**
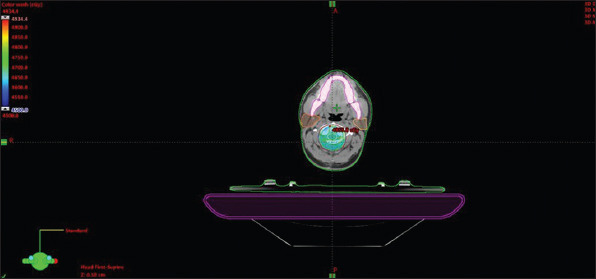
The treatment with rapid arc radiotherapy

A 27-year-old male with no known comorbidities was evaluated for neck pain and numbness in the right upper limb of 9 months’ duration. MRI of the entire neuroaxis revealed intramedullary hyperintense lesion at C2-C3 level showing patchy contrast enhancement. Clinical examination revealed neck stiffness, quadriparesis, and loss of sensation in the right upper limb. Sensations in the other three limbs were preserved. Deep tendon reflexes were brisk in all the four limbs and plantar reflex was flexor on both the lower limbs. He underwent laminectomy and partial excision of the lesion in another (private) hospital in August 2018. Postoperative MRI was suggestive of ill-defined partially hemorrhagic contrast-enhancing lesion in the cervical spinal cord at C2-C3 level with post-operative changes and residual lesion. The slide review in our institute was suggestive of GBM. The CSF cytology was negative. The patient was reviewed by the Neurosurgeon for any role of surgery. Since the lesion was adherent to the spinal cord and re-surgery might worsen the neurological status, surgery was deferred. After starting corticosteroids, he received radiotherapy of a total dose of 4500 cGy using Rapid Arc technique (Figures 2 and 3) along with concurrent temozolomide (75 mg/m^2^). The patient’s neurological status improved clinically with the above treatment and physiotherapy. He was then started on adjuvant temozolomide (150 mg/m^2^). After the first cycle of adjuvant temozolomide, he developed weakness in the left upper limb. Clinical examination revealed monoplegia in the left upper limb with loss of sensation. The MRI spine revealed diffuse spinal cord edema with disease progression. Since the patient’s condition started deteriorating rapidly, further chemotherapy was deferred. He was then started on intrathecal steroids as per the multidisciplinary board’s decision, but he succumbed to the illness due to the progressive nature of the disease.

### 2.3. Case 3

**Figure 4:**
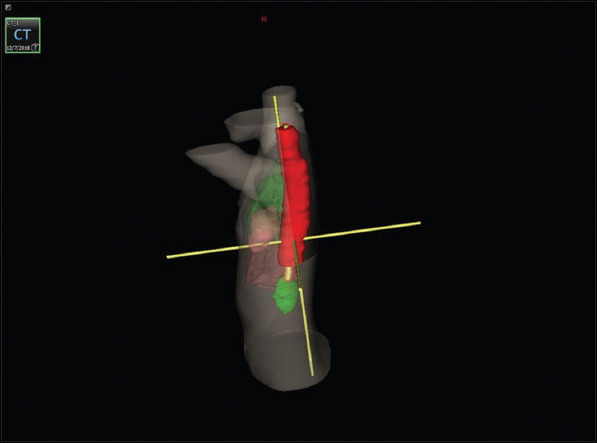
Three-dimensional plan of cervicothoracic lesion

**Figure 5:**
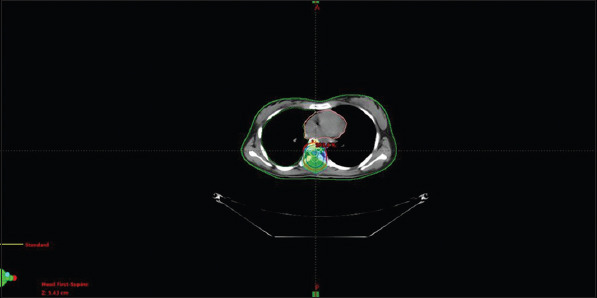
The treatment with conformal radiotherapy

A 13-year-old female was evaluated for difficulty in walking of 1-month duration which progressed to unsteadiness of gait. She then developed urinary incontinence for 3 days. The MRI of the entire neuroaxis was suggestive of diffuse enlargement of the cervicothoracic spinal cord from C6 to D8 with intramedullary solid and cystic expansile lesion with extensive edema. Clinical examination revealed that the muscle force in both upper limbs was 3/5 and in the lower limb was 0/5 (all the muscles). Sensory and deep tendon reflexes were absent on both the lower limbs. Plantar reflex showed Babinski’s sign on both the sides. She underwent C7-D7 laminectomy and excision of the lesion in November 2018. The post-operative histopathology was suggestive of GBM WHO grade IV. The CSF cytology was negative. The post-operative MRI showed post-operative changes with residual disease. Her neurological status did not improve much during the post-operative period. She received post-operative radiotherapy of a total dose of 4320 cGy using conformal technique (Figures 4 and 5) along with concurrent temozolomide (75 mg/m^2^). Since her parents were not willing for adjuvant temozolomide, further chemotherapy was deferred. Her neurological status improved, and she is on follow-up with a progression-free survival of 16 months without any evidence of acute or late radiation-related toxicities.

## 3. Discussion

Primary spinal GBM is very rare when compared to GBM of the brain probably due to less number of neuroglial cells in the spinal cord [4] and it occurs at a younger age probably in the second or third decades affecting both sexes [5]. In our case series, two patients were in the second and third decades, while the other was a pediatric patient.

The exact etiology of spinal GBM is unclear but may be attributed to radiation exposure [6]. The clinical presentation is variable depending on the segment and the extent of the spinal cord involvement. The common features are pain, weakness, sensory deficits, bowel, and bladder dysfunction. One or more of these features were present in all the patients reported in this case series. Contrast-enhanced MRI is the imaging modality of choice, which shows hypointense signals in T1-weighted images and hyperintense signals in T2-weighted images. Screening of the entire neuroaxis was done in all our patients to rule out primary spinal GBM as well as to identify drop metastasis, which is the routine protocol followed in our institute.

The diagnosis of GBM was established in our patients by clinical presentation, radiological features, and HPE including immunohistochemistry markers like glial fibrillary acidic protein, Ki-67, and S-100. Molecular testing was not done routinely in our institute during the study period, and hence, information on molecular analysis could not be obtained.

CSF metastasis can occur up to 26% in spinal GBM due to leptomeningeal dissemination which is attributed to relatively thin parenchyma in the spinal cord and short distance to the subarachnoid space [6]. Hence, CSF cytology is routinely done in spinal GBM.

Microscopically, GBM shows features like vascular proliferation, cellular pleomorphism, necrosis, and high mitotic activity similar to intracranial GBM [5]. The molecular studies show mutations of GBM are isocitrate dehydrogenase (IDH), 1p19q co-deletion, epidermal growth factor receptor (EGFR) amplification, and O-6-methylguanine-DNA methyl transferase (MGMT) promoter methylation [7]. GBM is an IDH wild-type tumor and carry EGFR amplification in around 45% and TERT mutations in around 80% of the patients [8,9].

When the MGMT promoter region is methylated in the tumor, patients with GBM show a better response with alkylating agents such as temozolomide [10]. Almost all the patients with an IDH mutation also show MGMT promoter methylation which is presumably due to the competitive inhibition of the alpha-ketoglutarate-dependent dioxygenase TET2 by the oncometabolite-2′-hydroxgluterate which results in a global hypermethylation of the genome of tumor cells with an IDH mutation occurs and the gCIMP genotype becomes detectable [11-13]. MGMT promoter is also subject to global hypermethylation and in the absence of an IDH mutation, MGMT promoter methylation is found in about 40% of all GBM patients. Since the above mutational studies were not done in our patients, exact molecular classification is not possible and that may be the reason for the longest survival in one of our patients of the case series.

There is no standard of care for spinal GBM. But the consensus of treatment in spinal GBM is surgery which includes maximal safe resection, followed by adjuvant radiotherapy and chemotherapy as per Stupp’s regimen, in line with GBM in the brain [14]. The mainstay of treatment and for obtaining the tissue diagnosis is maximal safe surgical resection. Several reports have shown that radiotherapy at a dose of 4000 cGy or higher was associated with a better outcome [15]. All our patients received radiotherapy to a total dose of more than 4000 cGy although the techniques were different (conformal and rapid arc radiotherapy). For gliomas with CSF positivity, craniospinal irradiation is usually indicated [15]. Since all our patients had negative CSF cytology, only focal radiation was given.

The role of temozolomide in primary spinal GBM is not well-established. Radiotherapy with concurrent temozolomide followed by adjuvant temozolomide improved the median survival of 16 months when compared to 9 months in other reports [3,15]. In our case series, only the first patient received the protocol treatment. Adjuvant temozolomide was deferred in the second and third patients due to progressive disease and parents’ unwillingness, respectively.

Intrathecal administration of beta interferon was used by Asano *et al*. as an adjunct to surgery and radiotherapy in the management of primary spinal GBM [16]. Immunotherapy with lymphokine-activated killer cells was used by Yamazaki *et al*. intrathecally and showed better outcome in a young woman treated with combination therapy [17]. As these are anecdotal evidence, one should be very cautious in using such agents.

Despite the multidisciplinary management, the prognosis of primary spinal GBM is grave. This may be attributed to the neurological deficits associated with radical surgery and high-dose radiotherapy. Hence, the surgery preferred is maximal safe surgical resection and the dose of radiotherapy is usually not exceeded beyond 5000 cGy to avoid radiation-induced myelitis. The median survival in various studies was only 15 months (6- 28 range). A rare case of prolonged survival of 64 months has been reported by Marchan *et al*.where cordectomy was performed in a paraplegic patient to delay the intracranial dissemination [18]. But the first patient of this case series has a better survival of 96 months. To the best of our knowledge, this patient has the longest survival quoted in the literature. None of the patients in the case series had acute or late radiation-related toxicities.

## 4. Conclusion

Since primary spinal GBM is a rare entity usually with a guarded prognosis, we wanted to highlight the case history of a patient with a better survival among the three cases treated in our institute. In addition to the multidisciplinary approach with surgery, radiotherapy, and chemotherapy, innovation of novel therapeutic approaches is needed for improving the management and outcome.

### Conflict of Interest

The authors declare no conflict of interest.
